# EVALUATING BIOLOGICAL AGE METRICS: ROBUST PREDICTORS OF AGING PROCESSES IN A CLINICAL COHORT

**DOI:** 10.1093/geroni/igae098.3657

**Published:** 2024-12-31

**Authors:** Elizabeth Stephens, Brianna Stubbs, Chatura Senadheera, Laura Alexander, Wendie Silverman-Martin, Sawyer Peralta, Stephanie Roa Diaz, John Newman

**Affiliations:** Buck Institute for Research on Aging, Rohnert Park, California, United States; Buck Institute, Novato, California, United States; Buck Institute, Novato, California, United States; Buck Institute, Novato, California, United States; Buck Institute, Novato, California, United States; Buck Institute for Research on Aging, Novato, California, United States; Buck Institute for Research on Aging, Novato, California, United States; Buck Institute for Research on Aging, Novato, California, United States

## Abstract

Biological age (BA) metrics, constructed by physical and biochemical markers, are increasingly recognized as powerful tools to quantify the biological processes associated with aging. Health and function in a population of the same chronological age (CA) varies between individuals. BA metrics, such as Klemera-Doubal Method (KDM), PhenoAge, and Homeostatic Dysregulation (HD) provide an age independent assessment of health status. Here, we aimed to examine the robustness and utility of BA metrics using data from a 12-week, randomized, parallel group, double-blind, placebo-controlled pilot study evaluating the safety and tolerability of a ketone ester (n=23; M=14, F=9; age=76y, range 65-90y). KDM and PhenoAge, and their residuals, showed strong correlations with CA (r=0.80 and r=0.89, respectively). In contrast, HD and its residual exhibited minimal correlation with CA (r=0.04). Using LASSO regression models, we identified several significant predictors of BA, including red cell distribution width, monocyte percentage, blood urea nitrogen, pulse and blood pressure, C-reactive protein, and total cholesterol and high-density lipoprotein. The models’ reliability was confirmed by minimizing prediction errors through cross-validated mean squared error paths, validating their robustness and generalizability. Our results validate KDM and PhenoAge as reliable BA metrics, emphasizing their potential use in clinical settings to assess aging and predict health outcomes. The biomarkers we found to predict BA reflect key biologic processes, such as inflammation, immune, kidney, and cardiac function, and metabolism, underscoring the relevance of BA as a complement to CA in assessing aging and age-related health risks.

